# MMP-12-mediated by SARM-TRIF signaling pathway contributes to IFN-γ-independent airway inflammation and AHR post RSV infection in nude mice

**DOI:** 10.1186/s12931-015-0176-8

**Published:** 2015-02-05

**Authors:** Xiaoru Long, Simin Li, Jun Xie, Wei Li, Na Zang, Luo Ren, Yu Deng, Xiaohong Xie, Lijia Wang, Zhou Fu, Enmei Liu

**Affiliations:** Ministry of Education Key Laboratory of Child Development and Disorders; Key Laboratory of Pediatrics in Chongqing, CSTC2009CA5002, Chongqing International Science and Technology Cooperation Center for Child Development and Disorders, Chongqing, 400014 P.R. China; Department of Respiratory Medicine, Children’s Hospital, Chongqing Medical University, No.136, Zhongshan 2nd Road, Yuzhong District, Chongqing, 400014 P.R. China

**Keywords:** RSV, MMP-12, SARM, TRIF, Airway inflammation, AHR

## Abstract

**Background:**

Respiratory syncytial virus (RSV) is one of the most frequently observed pathogens during infancy and childhood. However, the corresponding pathogenesis has not been determined to date. We previously demonstrated that IFN-γ plays an important role in RSV pathogenesis, and SARM-TRIF-signaling pathway could regulate the production of IFN-γ. This study is to investigate whether T cells or innate immune cells are the predominant producers of IFN-γ, and further to explore other culprits in addition to IFN-γ in the condition of RSV infection.

**Methods:**

Normal BALB/c mice and nude mice deficient in T cells were infected intranasally with RSV. Leukocytes in bronchoalveolar lavage fluid were counted, lung histopathology was examined, and airway hyperresponsiveness (AHR) was measured by whole-body plethysmography. IFN-γ and MMP-12 were detected by ELISA. MMP408, a selective MMP-12 inhibitor, was given intragastrically. Resveratrol, IFN-γ neutralizing antibody and recombinant murine IFN-γ were administered intraperitoneally. SARM and TRIF protein were semi-quantified by Western blot. siRNA was used to knock-down SARM expression.

**Results:**

RSV induced significant airway inflammation and AHR in both mice; IFN-γ was significantly increased in BALB/c mice but not in nude mice. MMP-12 was dramatically increased in both mice but earlier in nude mice. When MMP-12 was inhibited by MMP408, RSV-induced respiratory symptoms were alleviated. SARM was significantly suppressed while TRIF was significantly enhanced in both mice strains. Following resveratrol administration in nude mice, 1) SARM inhibition was prevented, 2) TRIF and MMP-12 were correspondingly down-regulated and 3) airway disorders were subsequently alleviated. Moreover, when SARM was efficiently knocked down using siRNA, TRIF and MMP-12 were markedly enhanced, and the anti-RSV effects of resveratrol were remarkably abrogated. MMP-12 was significantly increased in the IFN-γ neutralizing antibody-treated BALB/c mice but reduced in the recombinant murine IFN-γ-treated nude mice.

**Conclusions:**

MMP-12 can result in at least part of the airway inflammation and AHR independent of IFN-γ. And SARM-TRIF- signaling pathway is involved in regulating the overproduction of MMP-12. To the best of our knowledge, this study is the first that has examined the effects of SARM on MMP-12 and further highlights the potential to target SARM-TRIF-MMP-12 cascades to treat RSV infection.

**Electronic supplementary material:**

The online version of this article (doi:10.1186/s12931-015-0176-8) contains supplementary material, which is available to authorized users.

## Background

Respiratory syncytial virus (RSV) bronchiolitis in infants has long been a major public health and economic burden worldwide, particularly in low- and middle-income countries [1,2]. However, the development of efficient vaccines or antiviral medicines has been impeded by the as-yet contradictory pathogenic mechanisms [3].

We have demonstrated that IFN-γ is critical to the pathogenesis of RSV infection. Reducing IFN-γ by anti-IFN-γ antibody or resveratrol treatment significantly alleviated RSV-associated airway inflammation and airway hyper-responsiveness (AHR) [4,5]. Both CD4^+^ Th1 cells and CD8^+^ Tc1 cells contribute to the aberrant release of IFN-γ triggered by RSV [6-8], while innate immune cells, including NK cells and macrophages, among other cell types, are also essential sources [9,10]. However, which cell type is the primary producer of IFN-γ remains to be determined. Nude mice are congenitally deficient in T cells, but their innate immunity is normal or compensatorily enhanced [11]. Thus, we used this mouse model to investigate whether T cells or innate immune cells are the predominant producers of IFN-γ. RSV caused significant airway inflammation and AHR in nude mice, but unexpectedly, IFN-γ showed no perceivable changes throughout the disease in nude mice, which indicated that other non-T cells and non-IFN-γ proteins are involved.

In the absence of T cells, abundant macrophages were recruited into the airway of RSV-infected nude mice. It has been reported that macrophages can secrete MMP-12 in response to viral challenge [12]. MMP-12, also known as macrophage elastase, is a member of the matrix metalloproteinases (MMPs) family. The role of MMP-12 in asthma and COPD has been well-recognized [13,14]. Moreover, in preparing this manuscript, Foronjy and colleagues recently demonstrated that excessive lung protease (including MMP-12) responses were induced by RSV, and airway disorders could be alleviated by protease inhibitors [15]. When viewed in combination, it is reasonable to propose that MMP-12 might account for RSV-induced dysfunctions independent of T cells and IFN-γ.

Toll-like receptors (TLRs) and their down-stream adapter proteins are intimately associated with RSV infection. We demonstrated that sterile-α- and armadillo motif-containing protein (SARM), one of the adapter proteins, was suppressed by RSV [4]. SARM is a negative regulator of Toll/IL-1R domain-containing adapter inducing IFN-β (TRIF)-signaling cascades [16]. SARM suppression has consequently resulted in the over-expression of TRIF and IFN-γ and consequently resulted in RSV disease in BALB/c mice. Furthermore, resveratrol, a well-recognized antioxidant, could redress SARM-TRIF imbalance by up-regulating SARM, thereby reducing TRIF and IFN-γ, ultimately alleviating airway inflammation and AHR [4]. TLR signaling pathways also mediated the over-production of MMPs [17-19]. However, the role of SARM-TRIF disturbance in the exacerbation of MMP-12 has yet to be examined.

In the present study, we hypothesized that following RSV challenge, MMP-12 can be mediated by SARM-TRIF-signaling pathways similar to IFN-γ, and can result in airway inflammation and AHR independent of IFN-γ and T cells. Such studies will enhance our understanding of SARM-TRIF-signaling cascades and may help to identify new efficient strategies for the control of RSV infection.

## Methods

### Mice

In this study, six- to eight-week-old female BALB/c and nude mice (on a BALB/c background), free of specific pathogens, were purchased from the Animal Laboratory of Chongqing Medical University. The mice were bred under accredited specific pathogen-free conditions in separate filter-top cages according to the experimental design and were acclimated for at least one week prior to treatment. All experiments involving animals were in accordance with the Guide for the Care and Use of Laboratory Animals and approved by the Institutional Animal Care and Committee (IACUC), which is accredited by the Association for Assessment and Accreditation of Laboratory Animal Care International, China and Experimental Animal Committee of the Chongqing Medical University (license numbers: SCXK(Yu) 2012–0001 and SYXK(Yu) 2012–0001.

### RSV preparation and mice treatment

We utilized the A2 strain of human RSV (American Type Culture Collection). To inoculate RSV, the mice were held upright after sedation and inoculated intranasally with 1.5 × 10^7^ PFU RSV in a 100-μl volume or sham-infected with 100-μl cell culture supernatant (mock group). To assess the effect of MMP-12 on the airway inflammation and AHR, both BALB/c mice and nude mice were treated with MMP408, a potent and specific MMP-12 inhibitor, (CALBIOCHEM, EMD Chemicals, Inc. San Diego, CA 92121) at 5 mg/kg or PBS intragastrically twice a day from day 0 to day 8 post infection. Disease parameters were assessed on day 9. To assess the role of SARM-TRIF signaling pathway on MMP-12 regulation, nude mice were injected intraperitoneally with either resveratrol (Sigma-Aldrich Corp., St. Louis, MO) at 30 mg/kg/day [4] or PBS on day 0 (1 h post-RSV infection) and on days 1 to 4 consecutively. To assess the effects of IFN-γ on MMP-12 production, BALB/c mice were treated with IFN-γ neutralizing antibody (R4-6A2; BD PharMingen, San Diego, CA) and and nude mice were treated with recombinant murine IFN-γ (PeproTech, inc. Rocky Hill, NJ). IFN-γ neutralizing antibody (100 μg) and recombinant murine IFN-γ (10 μg) were injected intraperitoneally on day 0 (1 h post-RSV infection) and on days 1 and 3 post infection. Disease parameters were assessed on day 5. The corresponding isotype control antibodies were given similarly.

### Analysis of infiltrated inflammatory cells in BALF

Bronchoalveolar lavage fluid (BALF) was collected for cytokine concentration measurement and inflammatory cell evaluation as previously described [5]. Briefly, we lavaged the bronchial alveolar system of the mice with 0.5 ml ice-cold sterile PBS gently six times. The resultant BALF was centrifuged at 2500 rpm for 5 min at 4°C. Cell-free supernatant was aliquoted and stored at −80°C for subsequent cytokine detection. The remaining sediments were resuspended in 1 ml PBS. The total number of cells was quantified using microscopy. Cytospin slides were fixed and stained with DiffQuik (Baxter Healthcare Corp, Deerfield, Miami, FL) for leukocyte differential analysis. The number of neutrophils, macrophages, and lymphocytes in at least 200 cells per slide was quantified in a blind manner using a hemocytometer at × 1000 magnification.

### Morphological examinations

Mice were euthanized by cervical dislocation, and their left lung lobes were removed and fixed in 10% buffered formalin for 24 h. The lungs were then embedded in paraffin, cut into 5-mm-thick sections and stained with HE (hematoxylin and eosin) to evaluate RSV-associated pulmonary histopathology. To semi-quantitatively estimate lung lesions, a histopathological score (HPS) was performed as previously described [20]. The criteria assigned were 0 for no inflammation and 1, 2, and 3 for mild, moderate, and severe inflammation, respectively.

### RSV-induced AHR measurement

We used whole-body plethysmography (Emca instrument; Allmedicus, France) to assess airway hyperresponsiveness (AHR) at days 1, 3, 5 and 7 post-RSV inoculation. Briefly, conscious and spontaneously breathing mice were exposed to aerosolized phosphate-buffered saline (PBS) solution, followed by increasing concentrations of aerosolized methacholine solution (3.125, 6.25, 12.5, 25, 50 mg/ml; Sigma, USA) in PBS, for 3 min exposures. After each nebulization, there was a 2-min internal quiescent, and the enhanced pause (Penh) was calculated over the subsequent 5 min. Penh is a dimensionless parameter that represents pulmonary airflow resistance (Penh = PEP/PIP× pause).

### Determination of cytokines

The levels of IFN-γ and MMP-12 in BALF were measured using an enzyme-linked immunosorbent assay (ELISA) with commercial kits (Sizhengbai Beijing China) according to the manufacturer’s instructions. Duplicate wells were run, and the mean values were reported.

### Flow cytometry

Single-cell suspensions of lung were prepared and cells were incubated with PMA (50 ng/ml; Sigma), ionomycin (1000 ng/ml; Sigma) and GolgiPlug-containing brefeldin A (BD Biosciences) in 1 ml complete RPMI (RPMI 1640 medium supplemented with 10% fetal bovine serum, 2 mM L-glutamine, 100 U/ml penicillin and 100 μg/ml streptomycin). After 4–6 h incubation, the cells were harvested and blocked with antibody to mouse CD16/CD32 (Fc Block, BD). Samples were immunostained with antibody to mouse CD3, CD4, CD8 or isotype control conjugated with PerCP-cy5.5, FITC or PeCy7 for 30 minutes on ice and then fixed with 1% Formaldehyde in FACS Staining Buffer. The indicated antibodies were obtained from eBioscience (San Diego, CA). For intracellular IFN-γ detection, cells were fixed and permeabilized with CytoFix/CytoPerm solution (554722; BD) and Perm/Wash buffer (554723; BD) and then stained with APC-conjugated anti-IFN-γ mAb (BD Biosciences). Stained samples were measured on a flow cytometer, FACSCalibur (BD Biosciences). The data were analyzed using CellQuest software (BD Biosciences).

### Western blotting analysis

The total protein of lung tissues were extracted using a total protein extraction kit (KeyGEN, Nanjing, China). Samples containing equal quantities of protein were separated on an 8% SDS-PAGE (sodium dodecyl sulfate-polyacrylamide gel electrophoresis) gel and then transferred onto polyvinylidene difluoride (PVDF) membranes (Millipore, Billerica, MA). Membranes were probed with primary antibodies against SARM (1:500; SANTA, USA), TRIF (1:500; Abcam, Cambridge, MA) or β-actin (1:5,000; 4abio, Beijing, China). Alkaline phosphatase-conjugated goat anti-rabbit secondary antibody (1:10,000; MultiSciences, China) and goat anti-mouse antibody (1:10,000; MultiSciences, China) were used to detect the presence of the respective protein bands. Signals were quantified using Quantity One software (Bio-Rad, Hercules, CA) and normalized relative to β-actin.

### In vivo siRNA transfection

GFP-tagged siRNAs of SARM were purchased from Invitrogen (Shanghai, China) using the following sequences: SARM: 237868 (3–1); sense:TGCTGTGAAGAAGCGGCACAGTTTGTGTTTTGGCCACTGACTGACACAAACTGCCGCTTCTTCA; antisense:CCTGTGAAGAAGCGGCAGTTTGTGTCAGTCAGTGGCCAAAACACAAACTGTGCCGCTTCTTCAC; 237868 (Negative control): sense:tgctgAAATGTACTGCGCGTGGAGACGTTTTGGCCACTGACTGACGTCTCCACGCAGTACATTT, antisense:cctgAAATGTACTGCGTGGAGACGTCAGTCAGTGGCCAAAACGTCTCCACGCGCAGTACATTTc. We excluded the ectopic expression of SARM siRNA 3–1 in other organs and minimized its off-targets effects [4]. siRNA was dissolved in a solution of 5% glucose and in vivo jetPEI (Polyplus Transfection, New York, NY, USA) to an N/P ratio of 7 (number of nitrogen residues of jetPEI per RNA phosphate), and a total of 80 μL of siRNA-jetPEI complex was administered intranasally to nude mice on the fourth day post-infection. Disease parameters were assessed, and the samples were harvested at day 5 post-infection.

### Statistical analysis

All statistical tests were performed using Prism GraphPad Software (La Jolla, CA), and the results are expressed as the mean ± SEM. Two-way ANOVA with Bonferroni post-tests were used to compare the differences among multiple groups to AHR. Analysis of variance (ANOVA) was used to determine the differences between all groups to other indices. Data lacking normal distribution were evaluated using the nonparametric Kruskal-Wallis test, followed by Dunn’s multiple comparison. Differences were considered to be significant for p-values less than 0.05.

## Results

### RSV induces significant airway inflammation and AHR in BALB/c mice and nude mice

**Figure 1:**
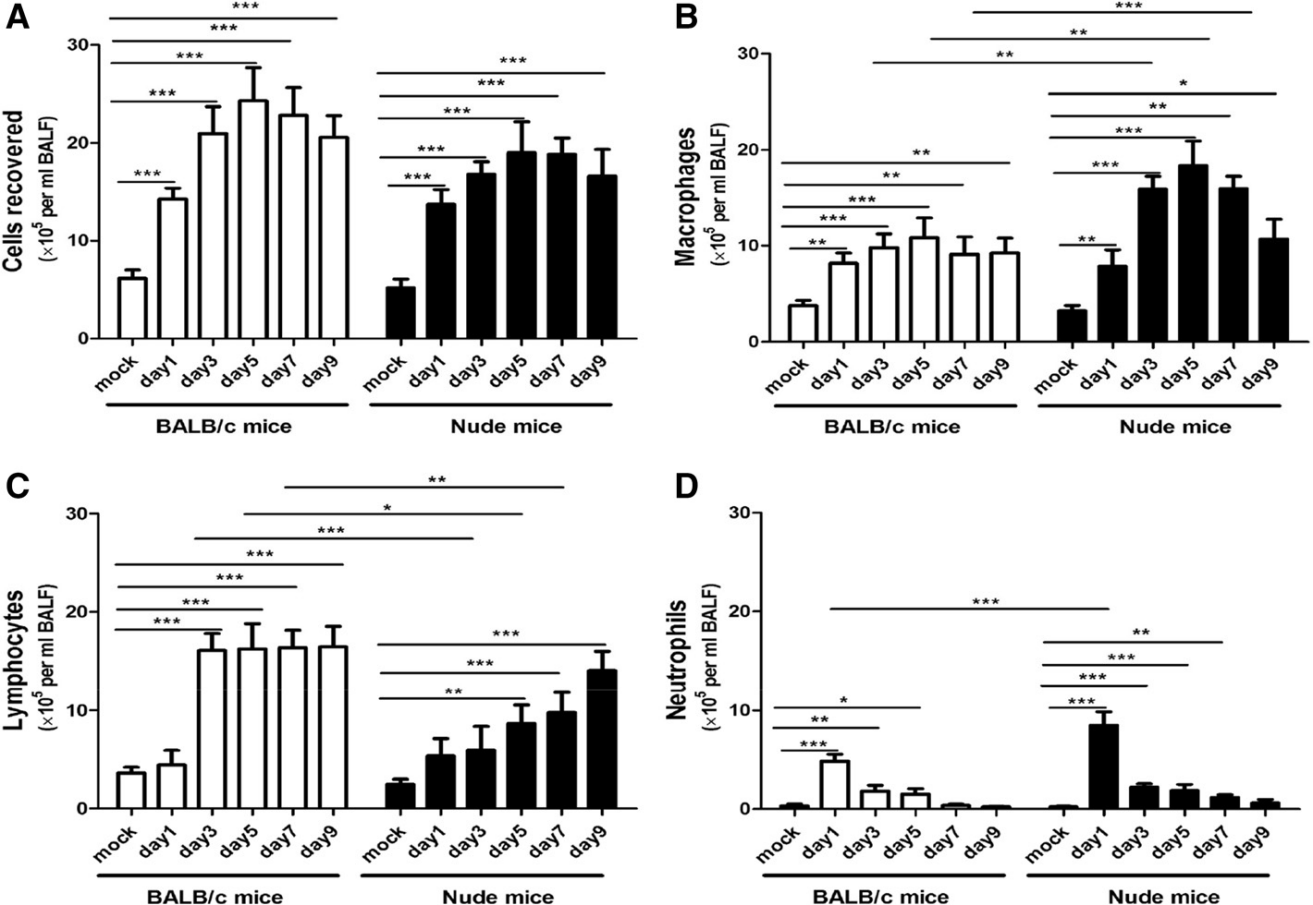
**RSV infection results in an increase in BALF cellularity.** Mice were infected with 1.5 × 10^7^ PFU of RSV and a group of animals were sacrificed at days 1, 3, 5, 7 and 9 post-infection. Total cells **(A)**, macrophages **(B)**, lymphocytes **(C)** and neutrophils **(D)** were all significantly increased following RSV challenge in both BALB/c mice and nude mice. Graphs are represented as the mean ± s.e.m. Data are representative of two independent experiments performed on 10 animals per group. *, p < 0.05, **, p < 0.01, ***, p < 0.001 shown comparing the corresponding mice groups are connected by a line.

In the absence of T cells, we found that RSV triggered significant airway inflammation and AHR in nude mice. As shown in Figure 1, abundant leukocytes (Figure 1A), including macrophages (Figure 1B), lymphocytes (Figure 1C) and neutrophils (Figure 1D) infiltrated into the bronchoalveolar lavage fluid (BALF) at days 1, 3, 5, 7 and 9 post-infection. Importantly, there were more macrophages and less lymphocytes in nude mice compared to BALB/c mice.

**Figure 2:**
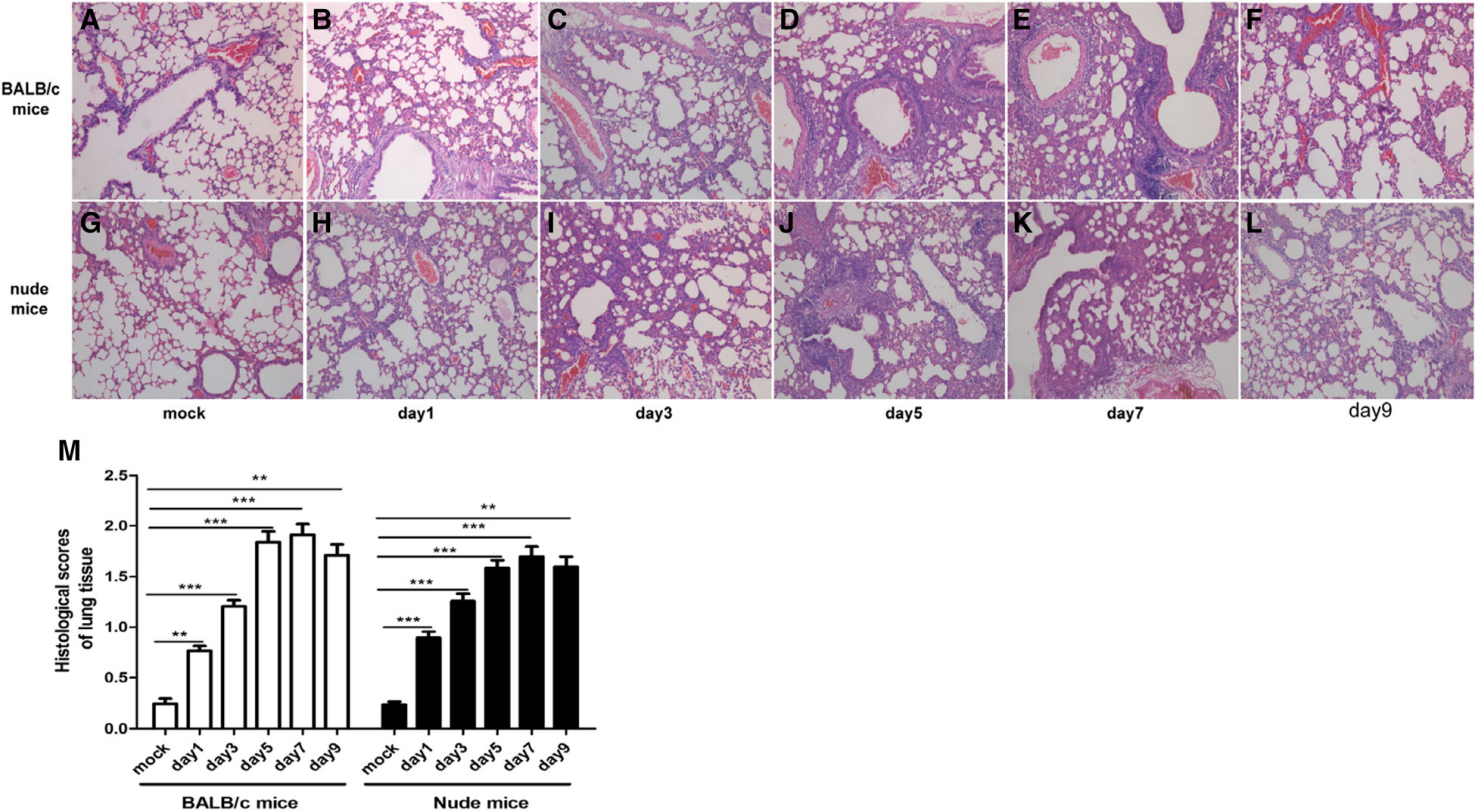
**RSV infection results in significant lung histopathological lesions.** Lungs were harvested at days 1, 3, 5, 7 and 9 post-RSV infection, stained with hematoxylin-and-eosin (HE), and scored for levels of inflammation as described in the Materials and Methods section. Representative HE staining of mouse lung tissue sections (original Magnification × 100) were shown. **A**: mock-infected BALB/c mice; **B**-**F**: RSV-infected BALB/c mice at days 1, 3, 5, 7 and 9 respectively. **G**: mock-infected nude mice; **H**-**L**: RSV-infected nude mice at days 1, 3, 5, 7 and 9 respectively. RSV induced a mass of inflammatory cells accumulation around the bronchiole, vascular and alveolar compartments, with goblet cell hyperplasia, mucus hypersecretion as well as partial destruction of regular tissue structures. **M**: Histological scores were remarkably elevated in the RSV-infected mice compared with the mock group. Graphs are representative of two independent experiments performed on 10 animals per group. *, p < 0.05, **, p < 0.01, ***, p < 0.001 shown comparing the corresponding mice groups are connected by a line.

Lung tissue damage is another parameter of RSV-associated dysfunction. As shown in Figure 2 A and G, no pulmonary hyper-cellularity or other pathological characteristics were observed in the mock-infected mice. However, at days 1 (Figure 2B and H), 3 (Figure 2C and I), 5 (Figure 2D and J), 7 (Figure 2E and K), and 9 (Figure 2F and L) post-infection, a mass of inflammatory cells accumulated around the bronchiole, vascular, and alveolar compartments, with goblet cell hyperplasia, mucus hypersecretion, and partial destruction of normal tissue structures in both BALB/c mice and nude mice. Corresponding to the morphological changes, the histological scores of lung tissues (Figure 2M) were significantly enhanced in RSV-challenged BALB/c mice and nude mice.

**Figure 3:**
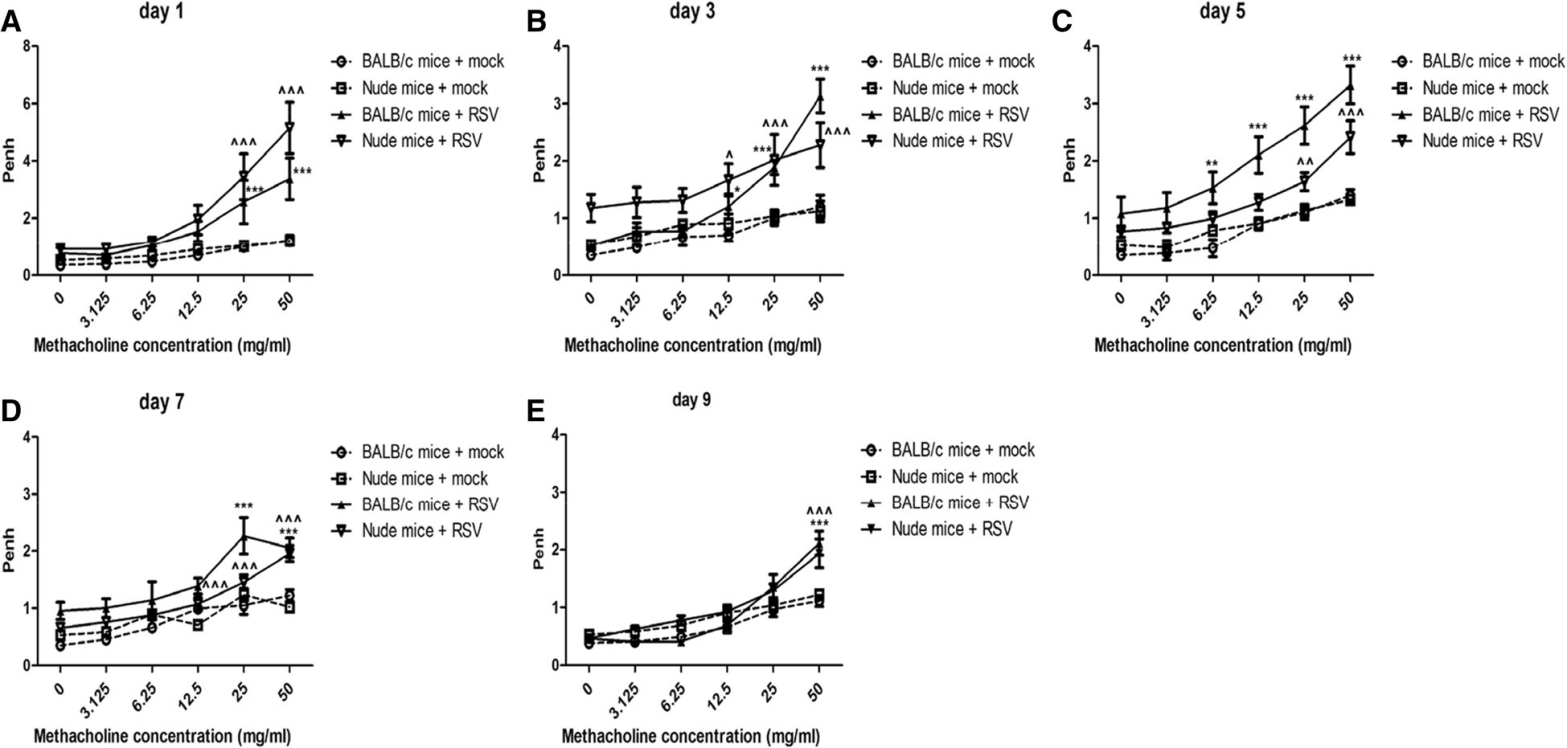
**RSV infection results in enhanced AHR.** Airway hyperresponsiveness to increasing doses of methacholine was assessed using plethysmography at days 1 **(A)**, 3 **(B)**, 5 **(C)**, 7 **(D)** and 9 **(E)** post-RSV infection. Penh values were significantly increased at all indicated time points following RSV challenge in both BALB/c mice and nude mice. Graphs are represented as the mean ± s.e.m. Data were representative of two independent experiments performed on 10 animals per group. *, p < 0.05, **, p < 0.01, ***, p < 0.001 for RSV-infected versus mock-treated BALB/c mice; ^, p < 0.05, ^^, p < 0.01, ^^^, p < 0.001 for RSV-infected versus mock-treated nude mice.

Next, we evaluated AHR caused by RSV and found that Penh values (enhanced pauses) were similarly elevated in BALB/c mice and nude mice at days 1, 3, 5, 7 and 9 post-infection (Figure 3A-E).

### T cells are the primary cellular sources of IFN-γ induced by RSV

**Figure 4:**
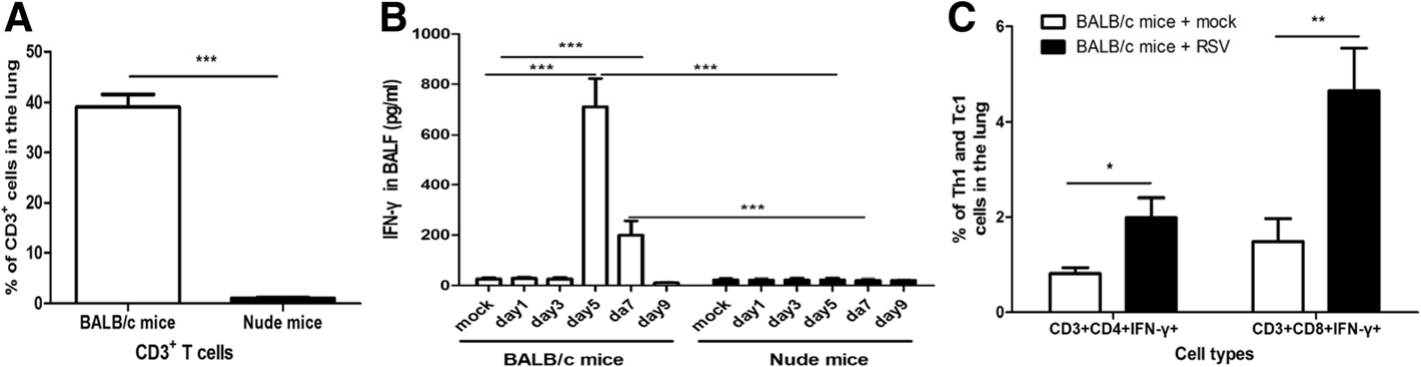
**IFN-γ** **induced by RSV is predominantly produced by T cells.** We confirmed that nude mice were deficient in CD3^+^ T cells **(A)** and found that IFN-γ was significantly increased at days 5 and 7 post-RSV infection in BALB/c mice, but was nearly negligible in nude mice **(B)**. We further found that both CD3^+^CD4^+^IFN-γ^+^ Th1 cells and CD3^+^CD8^+^IFN-γ^+^ Tc1 cells were remarkably elevated at day 5 when IFN-γ levels peaked in BALB/c mice **(C)**. Data are representative of two independent experiments performed on 6 animals per group. *, p < 0.05, **, p < 0.01, ***, p < 0.001 shown comparing the corresponding mice groups are connected by a line.

We and others have demonstrated the critical role of IFN-γ in the pathogenesis of RSV infection. Using flow cytometry, we confirmed that CD3^+^ T cells were nearly undetectable in nude mice (Figure 4A). Unexpectedly, IFN-γ showed no perceivable changes in nude mice throughout the assessed time points, but it was dramatically elevated in BALB/c mice at day 5 (p < 0.01) and day 7 (p < 0.05) post-infection (Figure 4B). In addition, IFN-γ was not exacerbated by RSV in NOD/SCID mice, which are deficient in both T cells and B cells (Additional file 1). Thus, IFN-γ could not be induced by RSV in the absence of T cells. We further demonstrated that CD3^+^CD4^+^IFN-γ^+^ Th1 and CD3^+^CD8^+^IFN-γ^+^ Tc1 cells were both significantly increased at day 5 when IFN-γ reached its peak level (Figure 4C). Taken together, these results indicated that T cells are the primary cellular sources of IFN-γ in the case of RSV infection; nevertheless, RSV can result in significant airway diseases independent of T cells and IFN-γ.

### MMP-12 contributes to RSV-induced resriratory symptoms

**Figure 5:**
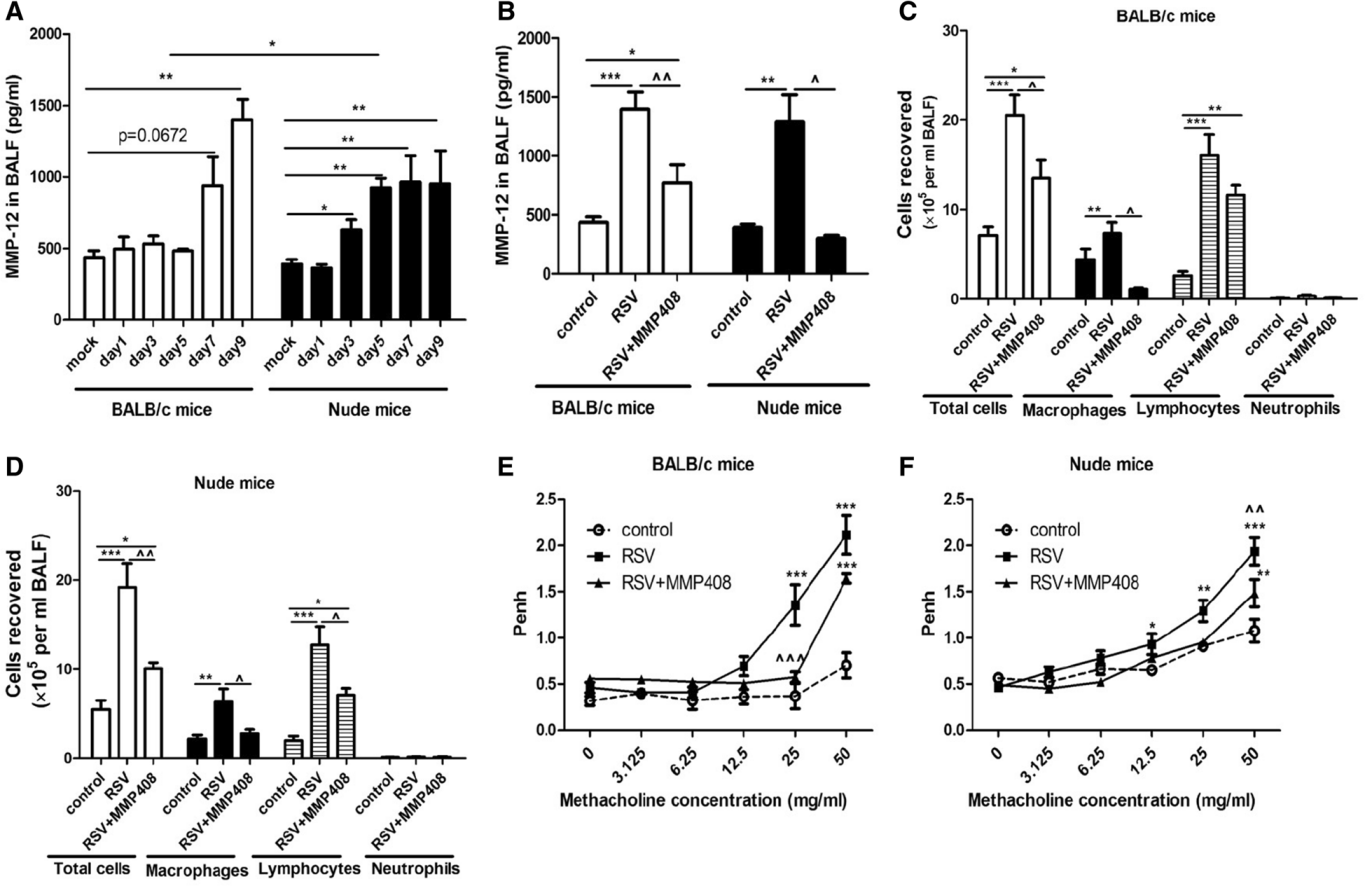
**MMP-12 contributes to RSV-induced resriratory symptoms.** Mice were killed at days 1, 3, 5, 7 and 9 post-infection, and MMP-12 was detected using ELISA. **A**: MMP-12 levels in BALF. **B**: MMP-12 levels following MMP408, a specific inhibitor of MMP-12,administration. **C**: Inflammatory cells in BALB/c mice. **D**: Inflammatory cells in nude mice. **E**: AHR in BALB/c mice. **F**: AHR in nude mice. mock: mock-infected mice group; control: mock-infected and PBS treated mice group; RSV: RSV-infected and PBS treated mice group; RSV + MMP408: RSV-infected and MMP408 treated mice group, Disease parameters were assessed on day 9 and data are representative of two independent experiments performed on 6–10 animals per group. *, p < 0.05, **, p < 0.01, ***, p < 0.001 shown comparing the control mice groups to other groups. ^, p < 0.05, ^^, p < 0.01, ^^^, p < 0.001 for RSV + PBS groups versus the RSV + MMP408 groups.

In search of the culprits that are responsible for airway disorders in the absence of T cells and IFN-γ, we found that MMP-12 levels were substantially increased in nude mice at days 3, 5, 7 and 9 post-infection. MMP-12 was not exacerbated in BALB/c mice on days 1, 3 and 5, whereas on day 7, MMP-12 tended to be elevated (p = 0.0672 vs. mock group), and on day 9, MMP-12 was significantly increased (Figure 5A). To further assess the effects of MMP-12 on airway inflammation and AHR caused by RSV, MMP408, a specific inhibitor of MMP-12 was administrated to mice. As shown in Figure 5B, MMP-12 was significantly blocked by MMP408. Moreover, airway inflammation (Figure 5C, D) and AHR (Figure 5E, F) of BALB/c mice and nude mice were both dramatically reduced in the MMP408-treated mice groups. Thus, MMP-12 can mediate at least part of the RSV-associated airway inflammation and AHR.

### SARM -TRIF signaling pathway is involved in MMP-12 production

In RSV-infected BALB/c mice, resveratrol treatment could significantly 1) suppress TRIF but prevent the RSV-mediated reduction of SARM expression; 2) decrease IFN-γ levels; and subsequently 3) reduce airway inflammatory cells and AHR (Additional file 2). Similar results have been published [4]. To examine the role of SARM-TRIF in MMP-12 modulation further, resveratrol and SARM siRNA were used to disrupt SARM-TRIF expression levels in nude mice. Nude mice were divided into five groups: control: mock-infected and PBS treated; RSV: RSV-infected and PBS treated; RSV + RES: RSV-infected and resveratrol treated; RSV + RES + 3-1: RSV-infected, resveratrol treated and SARM siRNA 3–1 administrated; RSV + RES + Negative: RSV-infected, resveratrol treated and negative siRNA vector administrated. Animals were killed at day 5 post-infection and all measurements were performed on this day.

**Figure 6:**
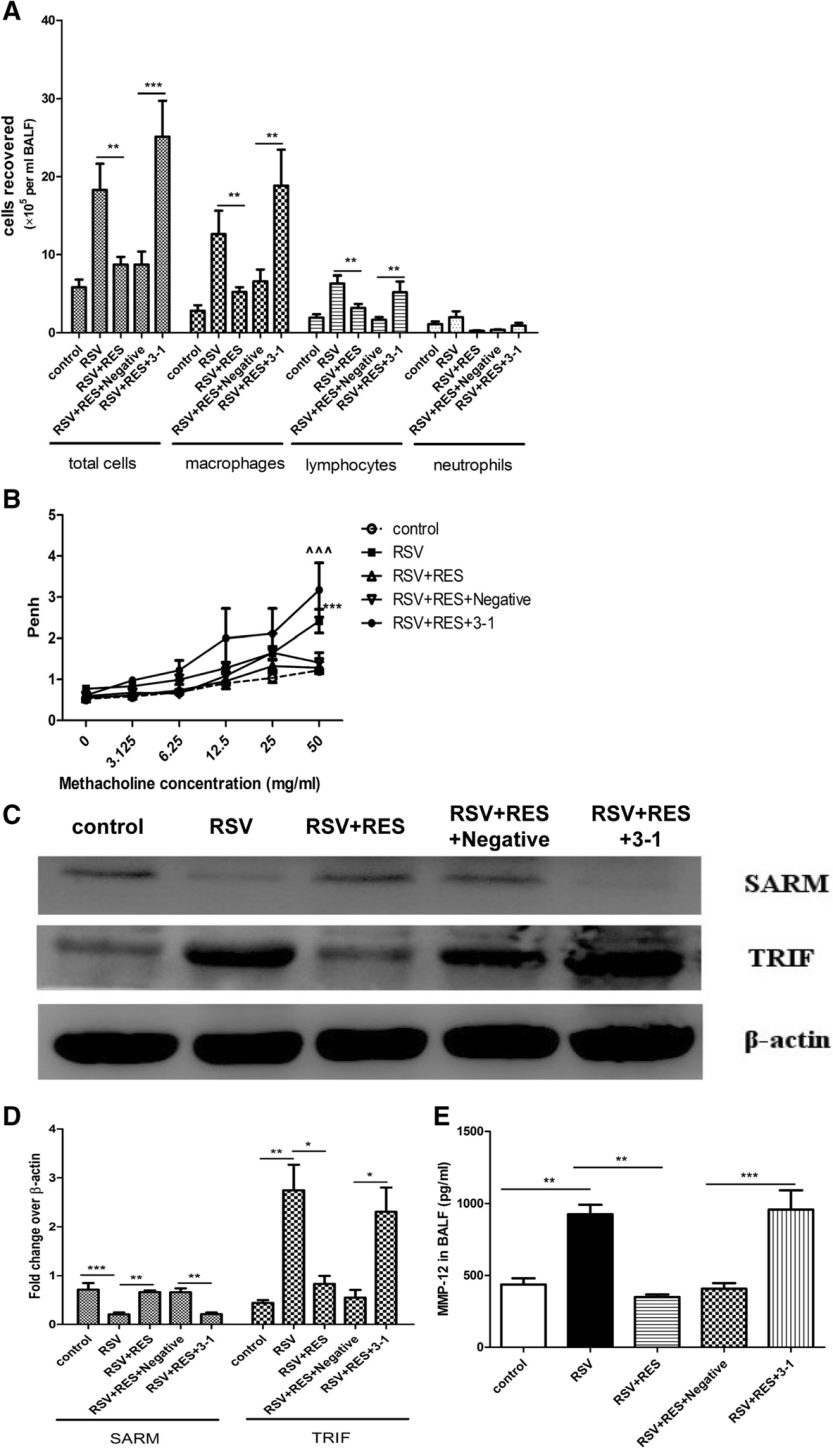
**SARM-TRIF signaling pathway is involved in regulating MMP-12 expression.** Nude mice were inoculated with RSV or mock infected with the culture supernatant. Resveratrol or PBS was injected intraperitoneally for 5 days consecutively. SARM siRNA 3–1 and the negative siRNA vector were transfected intranasally. The disease parameters were assessed at day 5 post-RSV infection. Nude mice were divided into five groups: control: mock-infected and PBS treated mice group; RSV: RSV-infected and PBS treated mice group; RSV + RES: RSV-infected and resveratrol treated mice group; RSV + RES + 3-1: RSV-infected, resveratrol treated and SARM siRNA 3–1 transfected mice group; RSV + RES + Negative: RSV-infected, resveratrol treated and negative siRNA vector administrated mice group. **A**: Inflammatory cells infiltrating into BALF. **B**: AHR in response to increasing doses of methacholine. **C**: Western blotting analysis of the expression of SARM and TRIF in the lung tissues. **D**: Semi-quantified expression of SARM and TRIF normalized to β-actin. E: MMP-12 protein levels in BALF. Data are representative of two independent experiments performed on 12 animals per group. In graph B, ***, p < 0.001 shown comparing the RSV group to the RSV + RES group; ^^^, p < 0.001 shown comparing the RSV + RES + 3-1 group to the RSV + RES + Negative group. In the other graphs, *, p < 0.05, **, p < 0.01, ***, p < 0.001 shown comparing the corresponding mice groups are connected by a line.

As shown in Figure 6A , airway infiltration of total inflammatory cells (p < 0.01), macrophages (p < 0.01) and lymphocytes (p < 0.01) was dramatically decreased in the RSV + RES group compared to the RSV group; and total inflammatory cells (p < 0.001), macrophages (p < 0.01) and lymphocytes (p < 0.01) were dramatically increased in the RSV + RES + 3-1 group compared to the RSV + RES + Negative group. As shown in Figure 6B, AHR of the RSV + RES group was almost equal to those of the control group, and AHR was significantly increased in the RSV + RES + 3-1 group compared to the RSV + RES + Negative group.

As shown in Figure 6C and D, SARM was significantly decreased (p < 0.001) while TRIF (P < 0.01) was significantly increased in the RSV group compared to the control group; following resveratrol administration, SARM inhibition was substantially prevented (p < 0.01), while TRIF (p < 0.05) was substantially down-regulated in the RSV + RES group compared to the RSV group. Moreover, when SARM expression was effectively knocked down by siRNA 3–1 (p < 0.01), the levels of TRIF (p < 0.05) was remarkably elevated in the RSV + RES + 3-1 group in contrast to the RSV + RES+ Negative group.

As shown in Figure 6E, MMP-12 was significantly decreased in the RSV + RES group compared to the RSV group (p < 0.01). Moreover, MMP-12 was significantly increased in the RSV + RES + 3-1 group compared to the RSV + RES+ Negative group. Taken together, these data indicated that SARM-TRIF signaling pathway is involved in regulating MMP-12 expression.

### IFN-γ suppresses MMP-12

**Figure 7:**
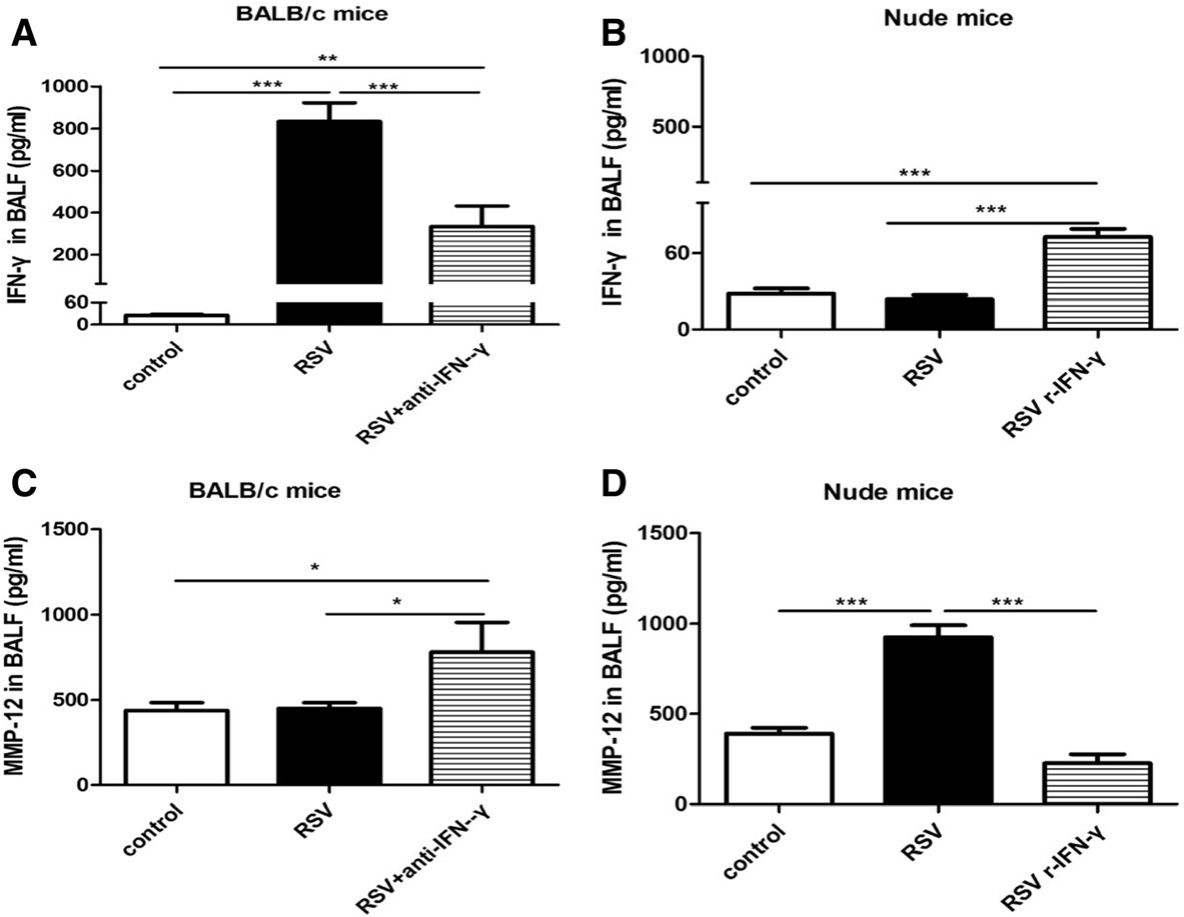
**IFN-γ** **suppresses MMP-12.** To assess the effects of IFN-γ on MMP-12 production in the condition of RSV challenge, BALB/c mice were treated with IFN-γ neutralizing antibody and nude mice were treated recombinant murine IFN-γ on days 0, 1, and 3 respectively. Samples were collected on day 5. **A**: IFN-γ levels in BALB/c mice. **B**: IFN-γ levels in nude mice. **C**: MMP-12 levels in BALB/c mice. **D**: MMP-12 levels in nude mice. control: mock-infected and PBS treated mice group; RSV + anti-IFN-γ: RSV-infected and IFN-γ neutralizing antibody treated BALB/c mice group; RSV + r-IFN-γ: RSV-infected and recombinant murine IFN-γ treated nude mice group, RSV: RSV-infected and isotype control antibodies treated BALB/c mice or nude mice groups. Data are representative of two independent experiments performed on 6–10 animals per group. *, p < 0.05, **, p < 0.01, ***, p < 0.001 shown comparing the the corresponding mice groups are connected by a line.

As mentioned above, MMP-12 was not significantly increased in RSV-infected BALB/c mice on day 5 when IFN-γ reached the peak, but tended to be increased on day 7 when IFN-γ began to decrease and significantly increased on day 9 when IFN-γ completely returned to baseline levels. Moreover, in the absence of IFN-γ in nude mice, MMP-12 was significantly increased on days 3, 5, 7 and 9. It was plausible to propose that IFN-γ might be able to suppress MMP-12 in the condition of RSV challenge. To confirm this, we treated BALB/c mice and nude mice with IFN-γ neutralizing antibody and recombinant murine IFN-γ respectively. IFN-γ levels were significantly decreased by neutralizing antibody treatment in BALB/c mice (Figure 7A), but were significantly increased by recombinant murine IFN-γ treatment in nude mice (Figure 7B). Moreover, MMP-12 was significantly increased in BALB/c mice treated with IFN-γ neutralizing antibody (Figure 7C), but was significantly decreased in nude mice treated with recombinant murine IFN-γ (Figure 7D).

## Discussion

In this study, we demonstrated that (1) T cells are the predominant cellular sources of IFN-γ induced by RSV; (2) MMP-12 accounts for at least part of the airway inflammation and AHR caused by RSV independent of IFN-γ; (3) SARM-TRIF signaling pathway is involved in regulating MMP-12 expression; and (4) IFN-γ can suppress MMP-12 expression in the condition of RSV challenge.

IFN-γ was significantly elevated in BALB/c mice, but not in nude mice or NOD/SCID mice, which are both deficient in T cells. Both CD3^+^CD4^+^IFN-γ^+^ Th1 cells and CD3^+^CD8^+^IFN-γ^+^ Tc1 cells were remarkably increased in BALB/c mice. These results strongly suggested that T cells are the primary producers of IFN-γ triggered by RSV. Our findings were consistent with recent evidence that in the PVM mouse model for human RSV, IFN-γ was undetectable until day 5 post-infection, at which time, CD8^+^ T cells infiltrated into the lung [7]. NK cells and macrophages and other cell types have been previously reported to be potential producers of IFN-γ [9,10]. Nevertheless, these conclusions were mainly drawn from mouse models with competent T cell responses, thus it cannot exclude the possibility that innate immune cells act as adjuvants of overt IFN-γ secretion by enhancing T cells activity. Indeed, activated NK cells are able to prime DCs to produce IL-12 and to induce highly protective CD8^+^ T cell memory responses [21]. However, Zhou *et al*. [22] have reported that nude mice have reduced IFN-γ levels on day 3 post RSV infection in contrast to BALB/c mice. The authors used 8–12 week old mice and they inoculated mice intranasally with 10^5^ PFU RSV in a volume of 50 μl. These differences might be responsible for part of the contradictions. In addition, the authors did not clarify the background of their nude mice model. Thus, the biological changes observed might also be background dependent.

In contrast to IFN-γ, MMP-12 was remarkably induced by RSV in both mice strains but earlier in nude mice. And when MMP-12 was suppressed by MMP408, airway inflammation and AHR were dramatically alleviated. Several previous studies have shown that RSV triggers dramatic up-regulation of lung proteases which can delay viral clearance and facilitate airway inflammation and AHR [12,15]. Foronjy *et al*. [15] have recently observed that MMP-12 was significantly increased on days 1, 3, 5, 7 and 9 in Friend leukemia virus B sensitive strain mice. Moreover, Marchant *et al*. [23] further reported that RSV challenge resulted in greater viral loads in MMP-12−/− mice compared to their wild-type counterparts. However, no researches have discussed the specific effects of MMP-12 on RSV pathogenesis to date. Our results clearly indicate that MMP-12 can lead to airway inflammation and AHR caused by RSV. Thus, therapies targeting MMP-12 may be promising anti-RSV options.

Cells of the monocyte-macrophage lineage are the largest source of MMP-12 in vivo and MMP-12 is critical for macrophages migration [24,25]. In nude mice, macrophages and MMP-12 were both significantly induced by RSV, and were both significantly reduced by resveratrol and MMP408 treatment. Our in vitro study has further shown that MMP-12 was dramatically increased in the RSV-challenged RAW 264.7 cells (Additional file 3). Thus, macrophages might contribute to the increased MMP-12 levels in our nude mice models, however, without specific cellular depletion, we can not identify the relationship of macrophages and MMP-12 in the present study.

Interestingly, although with a smaller magnitude, macrophages were also strongly induced in BALB/c mice on days 3, 5 and 7 following RSV challenge. Nevertheless, MMP-12 was not increased simultaneously. There are two possibilities for these divergences. First, macrophages might be phenotypically or functionally different in RSV-infected nude mice. Indeed, it has been demonstrated that in the absence of efficient lymphocyte and IFN-γ responses, macrophages failed to express a classically activated phenotype in response to RSV [26-28]. The second possibility is that MMP-12 was suppressed by IFN-γ. In our BALB/c mice models, MMP-12 tended to be increased on day 7 when IFN-γ began to decrease and was significantly increased on day 9 when IFN-γ completely returned to baseline levels. Moreover, after IFN-γ was neutralized in BALB/c mice, MMP-12 was significantly increased. And after IFN-γ was elevated by recombinant murine IFN-γ treatment in nude mice, MMP-12 was significantly decreased. It has been reported that IFN-γ can protect mice against aneurysm formation and blockade of IFN-γ signaling pathways resulting in abdominal aortic aneurysms primarily due to increased MMP-12 expression [29]. In addition, in vitro studies have also demonstrated that IFN-γ can inhibit MMP-9 in human monocytes and macrophages [30] and in murine peritoneal macrophages [31], as well as MMP-12 in murine macrophages [32].

The critical role of TLRs-SARM-TRIF-IFN-γ signaling pathways in the pathogenesis of RSV disease has been well-documented [4,5]. Our results further demonstrated that SARM-TRIF signaling pathway is involved in regulating MMP-12 expression. RSV infection significantly down-regulated SARM, and once SARM was restored by resveratrol treatment, the levels of MMP-12 were substantially reduced, which was accompanied by alleviated airway inflammation and AHR. In contrast, SARM knockdown almost completely reversed the anti-RSV effects of resveratrol. Foronjy *et al.* [15] indicated that TLR3-TRIF only partially regulated the protease response and RIG-I-MAVS exerted a far more substantial effect on airway proteases responses following RSV infection in Friend leukemia virus B sensitive strain mice. However, in our mice models, MAVS was significantly decreased (Additional file 4). These contradictions might be attributed to the mice strain differences. TLR3-TRIF has been recognized as important players in enhancing MMPs responses [19,33]. However, to the best of our knowledge, this is the first study to examine the effects of SARM on MMP-12.

## Conclusions

In summary, MMP-12 can result in at least part of the RSV-induced airway inflammation and AHR independent of IFN-γ and T cells. SARM-TRIF signaling pathways are critical for regulating RSV-induced airway inflammation and AHR, either by mediating overzealous T cells-IFN-γ responses or by mediating exacerbated macrophages-MMP-12 responses. These findings help to elucidate the complicated network of TLRs and lung proteases triggered by RSV and further highlight the potential to target SARM-TRIF- MMP-12 signaling cascades in the treatment of RSV infection.

## Additional files

Additional file 1:
**IFN-γ** **was not induced by RSV infection in NOD/SCID mice.** Six to eight weeks old NOD/SCID mice which are deficient in both T and B cells were inoculated intranasally with 1.5 × 10^7^ PFU RSV in a 100 μl volume or sham-infected with 100-μl cell culture supernatant (mock group). On day 5 post-infection, mice were killed and IFN-γ in BALF was detected using ELISA. IFN-γ was not increased in NOD/SCID mice on day 5. n = 4 in each group. Additional file 2:
**Effects of resveratrol treatment on RSV infection in BALB/c mice.** BALB/c mice were treated with resveratrol or PBS as described in the Materials and Methods section. Mice were divided into three groups: control: mock-infected and PBS treated; RSV: RSV-infected and PBS treated; RSV + RES: RSV-infected and resveratrol treated. Disease parameters were detected on day 5 post infection. A: Western blotting analysis of the expression of SARM and TRIF in the lung tissues. B: Semi-quantified expression of SARM and TRIF normalized to β-actin. C: IFN-γ levels in BALF. D: Inflammatory cells infiltrating into BALF. E: AHR in response to increasing doses of methacholine. n = 4. *, p < 0.05, **, p < 0.01, ***, p < 0.001 shown comparing the control mice groups to other groups.^, p < 0.05, ^^, p < 0.01, ^^^, p < 0.001 for RSV groups versus the RSV + RES groups. Additional file 3:
**MMP-12 was significantly increased in RSV-treated RAW264.7 cells.** The murine macrophage cell line RAW264.7 was purchased from ATCC and was cultured in Dulbecco’s Modified Eagle’s Medium (DMEM: GIBCO) at 37°C under 5% CO2. After an overnight culture, RAW264.7 cells in a 24-well plate was infected with RSV at a multiplicity of infection (MOI) of 0.5, 1 and 2 for 2 hours (h). To remove extracellular RSV, the cells were washed twice with 1 ml of PBS. The infection was allowed to continue for 24 h, 48 h, or 72 h. Supernatants were collected for MMP-12 detection at each time point. MMP-12 was significantly increased by RSV in the RAW 264.7 cells. *, p < 0.05, **, p < 0.01, ***, p < 0.001 shown comparing the corresponding groups are connected by a line. Additional file 4:
**MAVS was significantly suppressed by in RSV.** Lung tissues of both BALB/c mice and nude mice were harvested on day 7 post RSV infection. Total protein was extracted and western blot analyses were performed as described in the Materials and Methods section.The primary antibodies against MAVS (1:500; SANTA, USA), or β-actin (1:5,000; 4abio, Beijing, China) and Alkaline phosphatase-conjugated goat anti-rabbit secondary antibody (1:10,000; MultiSciences, China) and goat anti-mouse secondary antibody (1:10,000; MultiSciences, China) were used. MAVS was suppressed by RSV in both BALB/c mice (A) and nude mice (B).

## Abbreviations

AHR: Airway hyperresponsiveness
BALF: Bronchoalveolar lavage fluid
MMP-12: Matrix metalloproteinases-12
Penh: Enhanced pause
RES: Resveratrol
RSV: Respiratory syncytial virus
SARM: Sterile-and armadillo motif-containing protein
TRIF: TIR domain-containing adaptor inducing IFN-β-mediated transcription factor
TLRs: Toll-like receptors
