# Supplementary figures and images for: MMP-12-mediated by SARM-TRIF signaling pathway contributes to IFN-γ-independent airway inflammation and AHR post RSV infection in nude mice
Source: Respir Res. 2015 Feb 5;16(1):11. doi: 10.1186/s12931-015-0176-8 (PMC4332892; doi:10.1186/s12931-015-0176-8)

**Additional file 2**

**
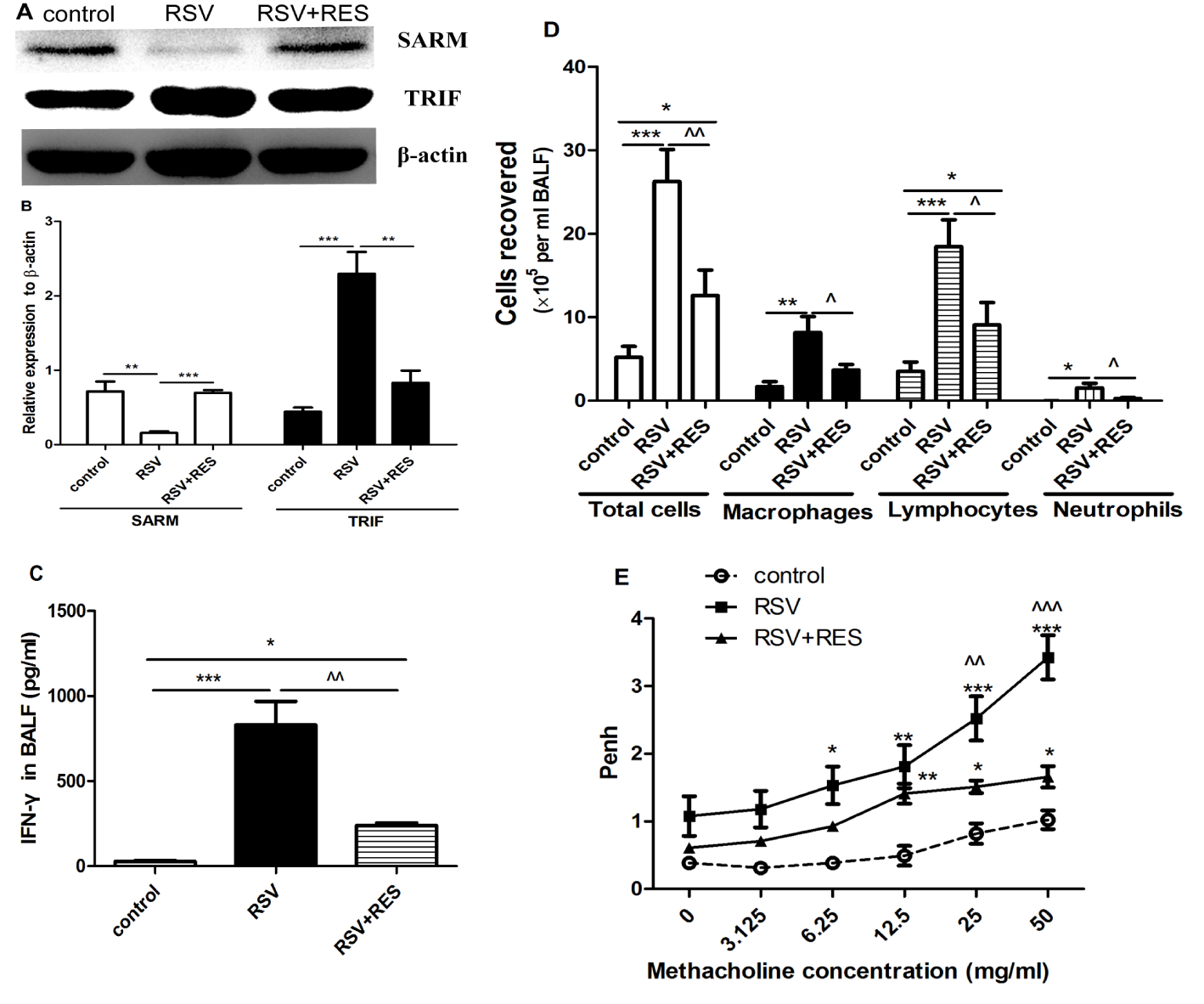
**

Supplement: Additional file 2: — Effects of resveratrol treatment on RSV infection in BALB/c mice. BALB/c mice were treated with resveratrol or PBS as described in the Materials and Methods section. Mice were divided into three groups: control: mock-infected and PBS treated; RSV: RSV-infected and PBS treated; RSV + RES: RSV-infected and resveratrol treated. Disease parameters were detected on day 5 post infection. A: Western blotting analysis of the expression of SARM and TRIF in the lung tissues. B: Semi-quantified expression of SARM and TRIF normalized to β-actin. C: IFN-γ levels in BALF. D: Inflammatory cells infiltrating into BALF. E: AHR in response to increasing doses of methacholine. n = 4. *, p < 0.05, **, p < 0.01, ***, p < 0.001 shown comparing the control mice groups to other groups.^, p < 0.05, ^^, p < 0.01, ^^^, p < 0.001 for RSV groups versus the RSV + RES groups. [file 12931_2015_176_MOESM2_ESM.doc]

**Additional file 4**

**
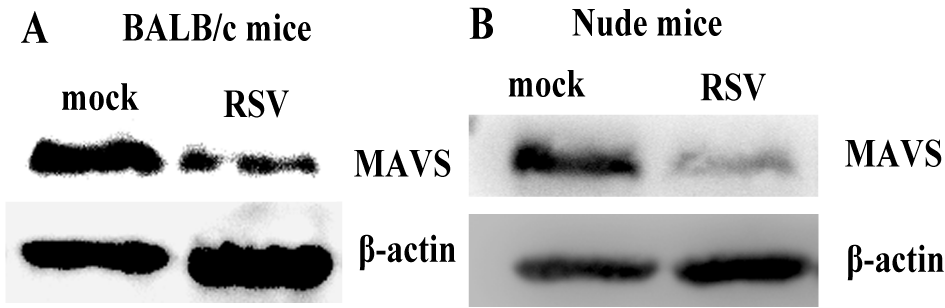
**

Supplement: Additional file 4: — MAVS was significantly suppressed by in RSV. Lung tissues of both BALB/c mice and nude mice were harvested on day 7 post RSV infection. Total protein was extracted and western blot analyses were performed as described in the Materials and Methods section.The primary antibodies against MAVS (1:500; SANTA, USA), or β-actin (1:5,000; 4abio, Beijing, China) and Alkaline phosphatase-conjugated goat anti-rabbit secondary antibody (1:10,000; MultiSciences, China) and goat anti-mouse secondary antibody (1:10,000; MultiSciences, China) were used. MAVS was suppressed by RSV in both BALB/c mice (A) and nude mice (B). [file 12931_2015_176_MOESM4_ESM.doc]
