# Supplementary material for: MMP-12-mediated by SARM-TRIF signaling pathway contributes to IFN-γ-independent airway inflammation and AHR post RSV infection in nude mice
Source: Respir Res. 2015 Feb 5;16(1):11. doi: 10.1186/s12931-015-0176-8 (PMC4332892; doi:10.1186/s12931-015-0176-8)
Supplement: Additional file 3: — MMP-12 was significantly increased in RSV-treated RAW264.7 cells. The murine macrophage cell line RAW264.7 was purchased from ATCC and was cultured in Dulbecco’s Modified Eagle’s Medium (DMEM: GIBCO) at 37°C under 5% CO2. After an overnight culture, RAW264.7 cells in a 24-well plate was infected with RSV at a multiplicity of infection (MOI) of 0.5, 1 and 2 for 2 hours (h). To remove extracellular RSV, the cells were washed twice with 1 ml of PBS. The infection was allowed to continue for 24 h, 48 h, or 72 h. Supernatants were collected for MMP-12 detection at each time point. MMP-12 was significantly increased by RSV in the RAW 264.7 cells. *, p < 0.05, **, p < 0.01, ***, p < 0.001 shown comparing the corresponding groups are connected by a line. [file 12931_2015_176_MOESM3_ESM.doc]

**Additional file 3**

**

**
